# Bis(2,3-di­chloro­phen­yl) di­sulfide

**DOI:** 10.1107/S1600536814007326

**Published:** 2014-04-09

**Authors:** Rebeca Nayely Osorio-Yáñez, Carmela Crisóstomo-Lucas, Ericka Santacruz-Juárez, Reyna Reyes-Martínez, David Morales-Morales

**Affiliations:** a Instituto de Química, Universidad Nacional Autónoma de México, Circuito exterior, Ciudad Universitaria, México, D.F., 04510, Mexico; b Universidad Politécnica de Tlaxcala, Av. Universidad Politécnica de Tlaxcala No. 1, San Pedro Xalcaltzinco Municipio de Tepeyanco, Tlaxcala, C.P. 90180, Mexico

## Abstract

The title compound, C_12_H_6_Cl_4_S_2_, features an S—S bond [2.0252 (8) Å] that bridges two 2,3-di­chloro­phenyl rings with a C—S—S—C torsion angle of 88.35 (11)°. The benzene rings are normal one to the other with a dihedral angle of 89.83 (11)°. The crystal structure features inter­molecular Cl⋯Cl [3.4763 (11) Å] and π–π stacking inter­actions [centroid–centroid distances = 3.696 (1) and 3.641 (2) Å]. Intra­molecular C—H⋯S inter­actions are also observed.

## Related literature   

For applications of di­sulfide compounds, see: Crowley (1964 ▶); Hashash *et al.* (2002 ▶); Gomez-Benitez *et al.* (2006 ▶); Yu *et al.* (2010 ▶). For various methods of synthesizing disulfides, see: Xiao *et al.* (2009 ▶); Shaabani *et al.* (2008 ▶); Ogilby (2010 ▶). For similar compounds and their crystal structures, see: Deng *et al.* (2003 ▶); Korp & Bernal (1984 ▶); Tang *et al.* (2011 ▶). For di­sulfide bonds in proteins, see: Sevier & Kaiser (2006 ▶). For van der Waals radii, see: Bondi (1964 ▶).
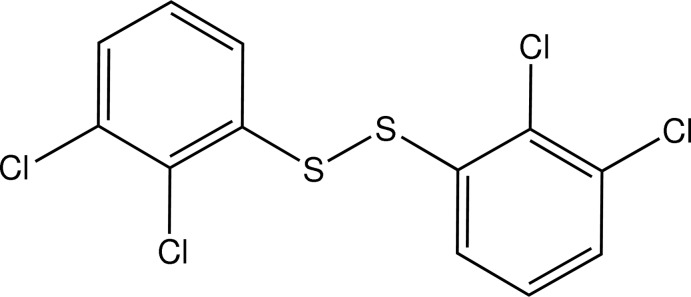


## Experimental   

### 

#### Crystal data  

C_12_H_6_Cl_4_S_2_
*M* *_r_* = 356.09
Triclinic, 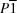

*a* = 7.7149 (10) Å
*b* = 7.7326 (11) Å
*c* = 12.748 (2) Å
α = 91.472 (2)°
β = 91.233 (3)°
γ = 114.859 (2)°
*V* = 689.37 (18) Å^3^
*Z* = 2
Mo *K*α radiation
μ = 1.14 mm^−1^
*T* = 298 K
0.37 × 0.24 × 0.14 mm

#### Data collection  

Bruker SMART APEX CCD diffractometer
Absorption correction: multi-scan (*SADABS*; Sheldrick, 2008 ▶) *T*_min_ = 0.678, *T*_max_ = 0.862
7044 measured reflections
3130 independent reflections
2594 reflections with *I* > 2σ(*I*)
*R*_int_ = 0.023

#### Refinement  

*R*[*F* ^2^ > 2σ(*F* ^2^)] = 0.035
*wR*(*F* ^2^) = 0.088
*S* = 1.03
3130 reflections
163 parameters
H-atom parameters constrained
Δρ_max_ = 0.41 e Å^−3^
Δρ_min_ = −0.30 e Å^−3^

### 

Data collection: *APEX2* (Bruker, 2012 ▶); cell refinement: *SAINT* (Bruker, 2012 ▶); data reduction: *SAINT*; program(s) used to solve structure: *SHELXS97* (Sheldrick, 2008 ▶); program(s) used to refine structure: *SHELXL97* (Sheldrick, 2008 ▶); molecular graphics: *ORTEP-3 for Windows* (Farrugia, 2012 ▶) and *DIAMOND* (Brandenburg, 2006 ▶); software used to prepare material for publication: *SHELXTL* (Sheldrick, 2008 ▶) and *PLATON* (Spek, 2009 ▶).

## Supplementary Material

Crystal structure: contains datablock(s) I. DOI: 10.1107/S1600536814007326/bx2456sup1.cif

Structure factors: contains datablock(s) I. DOI: 10.1107/S1600536814007326/bx2456Isup2.hkl

Supporting information file. DOI: 10.1107/S1600536814007326/bx2456Isup3.cml

CCDC reference: 994982

Additional supporting information:  crystallographic information; 3D view; checkCIF report

## Figures and Tables

**Table 1:** Hydrogen-bond geometry (Å, °)

*D*—H⋯*A*	*D*—H	H⋯*A*	*D*⋯*A*	*D*—H⋯*A*
C6—H6⋯S2	0.93	2.70	3.202 (2)	115
C12—H12⋯S1	0.93	2.70	3.199 (2)	115

**Fig. 1.**
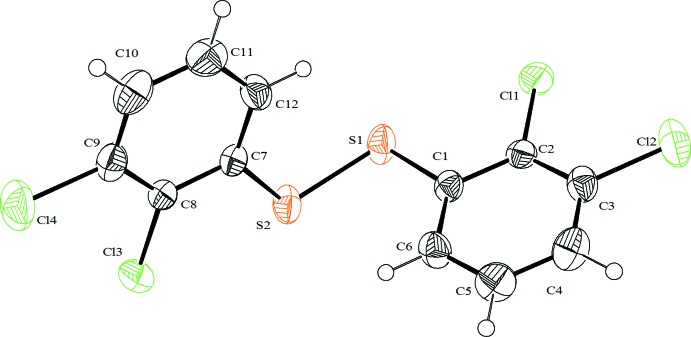
The molecular structure of the title compound showing 40% probability of displacement ellipsoids for the non-hydrogen atoms.

**Fig. 2.**
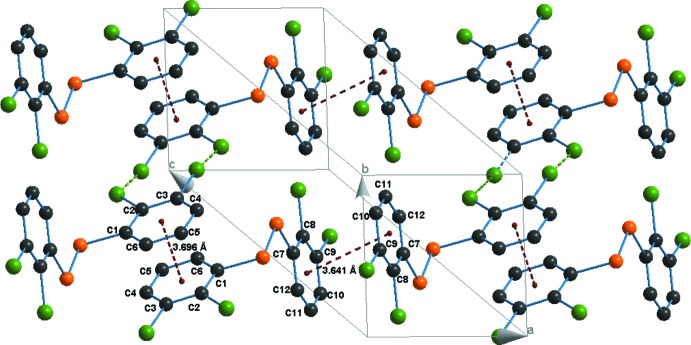
Representation of the π-π and Cl···Cl interactions shown by dashed lines. Hydrogen atoms are omitted.

## supplementary crystallographic information

### 1. Introduction

The di­sulfide bonds are found in proteins (Sevier and Kaiser, 2006), natural products and pharmacologically active compounds. Di­sulfide compounds have shown to exhibit activity as fungicide, mildew-proofing (Crowley, 1964) and anti­tumor agents (Hashash *et al.*, 2002). In organic synthesis di­sulfides are used in cross-coupling reactions catalyzed by transition metal compounds such as palladium, nickel and copper (Gomez-Benitez *et al.*, 2006; Yu *et al.*, 2010).

Several methods for the synthesis of di­sulfides have been reported. These processes involve the oxidative coupling of mercaptans by various oxidants such as molecular oxygen, nitric oxide, solvent-free permanganate, metal ions and promoted by sulfonyl chloride in aqueous media (Xiao *et al.*, 2009; Shaabani *et al.*, 2008; Ogilby, 2010).

Thus, in this report we present the crystal structure of the bis­(2,3-di­chloro­phenyl)­disulfide obtained by a nucleophilic substitution reaction. The structure is represented in figure 1.

### 2.1. Synthesis and crystallization

The title compound was obtained as a by-product of the reaction between 2-(chloro­methyl)­benzimidazole (0.2 g) and the lead salt of 2,3-di­chloro­benze­thiol ([Pb(SC_6_H_3_-2,3-Cl_2_)_2_]) (0.337 g) in toluene. The resulting reaction mixture was allowed to proceed under reflux by 8 h after which time the formation of PbCl_2_ was observed indicating completion of the reaction. The reaction mixture was then filtered through a short Celite plug to afford a colorless solution, the solvent was evaporated under vacuum and the residue column chromatographed (silica gel 60, eluted with 3/2 ethyl acetate/hexane system). Slow Evaporation of the first fraction collected produced crystals of the title compound suitable for X-ray diffraction analysis.

### 2.2. Refinement

Crystal data, data collection and structure refinement details are summarized in Table 1.

H atoms were included in calculated positions (C—H = 0.93 A for aromatic H) and refined using a riding model with *U*iso(H) = 1.2 *U*eq of the carrier atoms.

In the refinement six reflections, (2 0 0), (0 1 3), (0 0 1), (-2 1 2), (2 -4 3) and (-1 0 1), were considered as disagreeable and were omitted.

### 3. Results and discussion

The asymmetric unit of the title compound consists of one molecule on the di­sulfide. The rings of the bis­(2,3-di­chloro­phenyl)­disulfide show a dihedral angle of 89.83° between the two planes and a torsion angle C1—S1—S2—C7 of 88.35 (11)°. The value of the C—S—S—C torsion angle is similar to those found in similar compounds, such as bis­(penta­chloro­phenyl)­disulfide (Deng *et al.*, 2003), di­phenyl­disulfide (Korp & Bernal, 1984) and bis­(4-amino-2-chloro­phenyl)­disulfide (Tang *et al.*, 2011). The S—S distance is 2.0252 (8) Å, whereas the C—S distances are 1.784 (2) and 1.7835 (19) Å. These values are similar and close in value to compounds such as bis­(penta­chloro­phenyl)­disulfide with a S—S distance of 2.063 (2) Å and bis­(4-amino-2-chloro­phenyl)­disulfide of 2.0671 (16) Å. The crystal packing is stabilized by π-π and Cl···Cl inter­actions (Figure 2). The π-π inter­actions of the 2,3-di­chloro­phenyl rings presents distances between centroids of 3.696 (1) and 3.641 (2) Å. The Cl1···Cl2 contact distance is of 3.476 Å that is close to the sum of the van der Waals radii of the chloride atoms (Bondi, 1964). The sulphur atoms present C—H···S intra­molecular inter­actions, these values are in the table 1.

### Crystal data

**Table:**

C_12_H_6_Cl_4_S_2_	*Z* = 2
*M**_r_* = 356.09	*F*(000) = 356
Triclinic, *P*1	*D*_x_ = 1.715 Mg m^−^^3^
*a* = 7.7149 (10) Å	Mo *K*α radiation, λ = 0.71073 Å
*b* = 7.7326 (11) Å	Cell parameters from 4288 reflections
*c* = 12.748 (2) Å	θ = 2.8–27.5°
α = 91.472 (2)°	µ = 1.14 mm^−^^1^
β = 91.233 (3)°	*T* = 298 K
γ = 114.859 (2)°	Prism, colourless
*V* = 689.37 (18) Å^3^	0.37 × 0.24 × 0.14 mm

### Data collection

**Table:**

Bruker SMART APEX CCD diffractometer	3130 independent reflections
Radiation source: fine-focus sealed tube	2594 reflections with *I* > 2σ(*I*)
Graphite monochromator	*R*_int_ = 0.023
Detector resolution: 8.333 pixels mm^-1^	θ_max_ = 27.5°, θ_min_ = 2.9°
ω scans	*h* = −9→9
Absorption correction: multi-scan (*SADABS*; Sheldrick, 2008)	*k* = −10→10
*T*_min_ = 0.678, *T*_max_ = 0.862	*l* = −16→16
7044 measured reflections	

### Refinement

**Table:**

Refinement on *F*^2^	Primary atom site location: structure-invariant direct methods
Least-squares matrix: full	Secondary atom site location: difference Fourier map
*R*[*F*^2^ > 2σ(*F*^2^)] = 0.035	Hydrogen site location: inferred from neighbouring sites
*wR*(*F*^2^) = 0.088	H-atom parameters constrained
*S* = 1.03	*w* = 1/[σ^2^(*F*_o_^2^) + (0.0397*P*)^2^ + 0.1749*P*] where *P* = (*F*_o_^2^ + 2*F*_c_^2^)/3
3130 reflections	(Δ/σ)_max_ < 0.001
163 parameters	Δρ_max_ = 0.41 e Å^−^^3^
0 restraints	Δρ_min_ = −0.30 e Å^−^^3^

### Special details

**Table:**

Geometry. All e.s.d.'s (except the e.s.d. in the dihedral angle between two l.s. planes) are estimated using the full covariance matrix. The cell e.s.d.'s are taken into account individually in the estimation of e.s.d.'s in distances, angles and torsion angles; correlations between e.s.d.'s in cell parameters are only used when they are defined by crystal symmetry. An approximate (isotropic) treatment of cell e.s.d.'s is used for estimating e.s.d.'s involving l.s. planes.

### Fractional atomic coordinates and isotropic or equivalent isotropic displacement parameters (Å^2^)

**Table:**

	*x*	*y*	*z*	*U*_iso_*/*U*_eq_	
Cl1	0.81619 (10)	0.72799 (9)	0.12199 (5)	0.06495 (19)	
Cl2	1.23381 (10)	0.95252 (8)	0.04969 (5)	0.0714 (2)	
Cl3	0.64782 (10)	−0.26178 (7)	0.37441 (5)	0.06220 (18)	
Cl4	0.75120 (11)	−0.22491 (11)	0.61516 (5)	0.0772 (2)	
S1	0.67633 (8)	0.32903 (8)	0.20430 (5)	0.05302 (16)	
S2	0.64526 (8)	0.07120 (7)	0.25388 (4)	0.05061 (16)	
C1	0.9189 (3)	0.4447 (3)	0.16702 (13)	0.0369 (4)	
C2	0.9766 (3)	0.6267 (3)	0.12843 (13)	0.0372 (4)	
C3	1.1620 (3)	0.7266 (3)	0.09688 (15)	0.0433 (4)	
C4	1.2912 (3)	0.6466 (3)	0.10343 (18)	0.0539 (5)	
H4	1.4159	0.7138	0.0825	0.065*	
C5	1.2338 (3)	0.4663 (3)	0.14126 (18)	0.0548 (5)	
H5	1.3207	0.4119	0.1455	0.066*	
C6	1.0495 (3)	0.3650 (3)	0.17301 (16)	0.0462 (5)	
H6	1.0129	0.2434	0.1984	0.055*	
C7	0.7117 (3)	0.1092 (3)	0.39027 (14)	0.0364 (4)	
C8	0.7107 (3)	−0.0468 (3)	0.44284 (14)	0.0378 (4)	
C9	0.7575 (3)	−0.0306 (3)	0.54892 (16)	0.0447 (5)	
C10	0.8069 (3)	0.1407 (4)	0.60354 (17)	0.0564 (6)	
H10	0.8395	0.1517	0.6748	0.068*	
C11	0.8078 (3)	0.2947 (3)	0.55208 (18)	0.0561 (6)	
H11	0.8405	0.4099	0.5891	0.067*	
C12	0.7608 (3)	0.2809 (3)	0.44603 (17)	0.0460 (5)	
H12	0.7620	0.3864	0.4121	0.055*	

### Atomic displacement parameters (Å^2^)

**Table:**

	*U*^11^	*U*^22^	*U*^33^	*U*^12^	*U*^13^	*U*^23^
Cl1	0.0859 (4)	0.0712 (4)	0.0671 (4)	0.0592 (3)	0.0296 (3)	0.0276 (3)
Cl2	0.0806 (4)	0.0444 (3)	0.0751 (4)	0.0115 (3)	0.0078 (3)	0.0195 (3)
Cl3	0.0915 (5)	0.0416 (3)	0.0596 (4)	0.0333 (3)	0.0146 (3)	0.0022 (2)
Cl4	0.0910 (5)	0.0889 (5)	0.0677 (4)	0.0512 (4)	0.0099 (3)	0.0389 (3)
S1	0.0447 (3)	0.0628 (3)	0.0570 (3)	0.0262 (3)	0.0104 (2)	0.0284 (3)
S2	0.0609 (3)	0.0430 (3)	0.0377 (3)	0.0117 (2)	−0.0002 (2)	0.0080 (2)
C1	0.0414 (10)	0.0419 (9)	0.0290 (9)	0.0188 (8)	0.0000 (7)	0.0059 (7)
C2	0.0487 (11)	0.0412 (9)	0.0274 (9)	0.0246 (9)	0.0011 (7)	0.0022 (7)
C3	0.0511 (12)	0.0395 (10)	0.0329 (9)	0.0128 (9)	−0.0014 (8)	0.0030 (7)
C4	0.0364 (11)	0.0659 (14)	0.0526 (13)	0.0148 (10)	−0.0018 (9)	0.0069 (11)
C5	0.0465 (12)	0.0707 (14)	0.0576 (13)	0.0348 (11)	−0.0038 (10)	0.0085 (11)
C6	0.0476 (11)	0.0481 (11)	0.0481 (11)	0.0251 (9)	−0.0026 (9)	0.0112 (9)
C7	0.0341 (9)	0.0373 (9)	0.0347 (9)	0.0116 (7)	0.0058 (7)	0.0057 (7)
C8	0.0383 (10)	0.0372 (9)	0.0396 (10)	0.0168 (8)	0.0093 (8)	0.0062 (7)
C9	0.0377 (10)	0.0543 (12)	0.0433 (11)	0.0197 (9)	0.0065 (8)	0.0144 (9)
C10	0.0462 (12)	0.0723 (15)	0.0395 (11)	0.0141 (11)	−0.0012 (9)	0.0010 (10)
C11	0.0520 (13)	0.0469 (12)	0.0547 (13)	0.0070 (10)	0.0028 (10)	−0.0125 (10)
C12	0.0456 (11)	0.0338 (9)	0.0536 (12)	0.0115 (8)	0.0046 (9)	0.0054 (8)

### Geometric parameters (Å, º)

**Table:**

Cl1—C2	1.7224 (19)	C5—C6	1.380 (3)
Cl2—C3	1.725 (2)	C5—H5	0.9300
Cl3—C8	1.7291 (19)	C6—H6	0.9300
Cl4—C9	1.726 (2)	C7—C12	1.389 (3)
S1—C1	1.784 (2)	C7—C8	1.392 (3)
S1—S2	2.0252 (8)	C8—C9	1.381 (3)
S2—C7	1.7834 (19)	C9—C10	1.378 (3)
C1—C6	1.386 (3)	C10—C11	1.372 (3)
C1—C2	1.393 (2)	C10—H10	0.9300
C2—C3	1.384 (3)	C11—C12	1.382 (3)
C3—C4	1.378 (3)	C11—H11	0.9300
C4—C5	1.378 (3)	C12—H12	0.9300
C4—H4	0.9300		
			
C1—S1—S2	105.09 (7)	C1—C6—H6	120.0
C7—S2—S1	105.02 (7)	C12—C7—C8	119.02 (17)
C6—C1—C2	119.06 (18)	C12—C7—S2	124.42 (15)
C6—C1—S1	124.55 (15)	C8—C7—S2	116.55 (14)
C2—C1—S1	116.38 (14)	C9—C8—C7	120.32 (17)
C3—C2—C1	120.36 (18)	C9—C8—Cl3	120.28 (15)
C3—C2—Cl1	120.22 (14)	C7—C8—Cl3	119.39 (14)
C1—C2—Cl1	119.42 (15)	C10—C9—C8	120.27 (19)
C4—C3—C2	120.23 (18)	C10—C9—Cl4	119.19 (17)
C4—C3—Cl2	119.41 (17)	C8—C9—Cl4	120.53 (16)
C2—C3—Cl2	120.36 (16)	C11—C10—C9	119.6 (2)
C5—C4—C3	119.4 (2)	C11—C10—H10	120.2
C5—C4—H4	120.3	C9—C10—H10	120.2
C3—C4—H4	120.3	C10—C11—C12	120.9 (2)
C4—C5—C6	121.0 (2)	C10—C11—H11	119.5
C4—C5—H5	119.5	C12—C11—H11	119.5
C6—C5—H5	119.5	C11—C12—C7	119.85 (19)
C5—C6—C1	119.93 (19)	C11—C12—H12	120.1
C5—C6—H6	120.0	C7—C12—H12	120.1
			
S2—S1—C1—C6	0.06 (18)	S1—S2—C7—C12	3.60 (18)
S2—S1—C1—C2	179.34 (12)	S1—S2—C7—C8	−177.28 (13)
C6—C1—C2—C3	−0.2 (3)	C12—C7—C8—C9	0.1 (3)
S1—C1—C2—C3	−179.52 (14)	S2—C7—C8—C9	−179.02 (14)
C6—C1—C2—Cl1	−179.31 (14)	C12—C7—C8—Cl3	179.45 (14)
S1—C1—C2—Cl1	1.4 (2)	S2—C7—C8—Cl3	0.3 (2)
C1—C2—C3—C4	0.0 (3)	C7—C8—C9—C10	−0.5 (3)
Cl1—C2—C3—C4	179.07 (16)	Cl3—C8—C9—C10	−179.76 (16)
C1—C2—C3—Cl2	−179.68 (14)	C7—C8—C9—Cl4	178.52 (14)
Cl1—C2—C3—Cl2	−0.6 (2)	Cl3—C8—C9—Cl4	−0.8 (2)
C2—C3—C4—C5	0.3 (3)	C8—C9—C10—C11	0.6 (3)
Cl2—C3—C4—C5	179.91 (17)	Cl4—C9—C10—C11	−178.44 (17)
C3—C4—C5—C6	−0.3 (3)	C9—C10—C11—C12	−0.3 (3)
C4—C5—C6—C1	0.0 (3)	C10—C11—C12—C7	0.0 (3)
C2—C1—C6—C5	0.2 (3)	C8—C7—C12—C11	0.1 (3)
S1—C1—C6—C5	179.46 (16)	S2—C7—C12—C11	179.18 (16)

### Hydrogen-bond geometry (Å, º)

**Table:**

*D*—H···*A*	*D*—H	H···*A*	*D*···*A*	*D*—H···*A*
C6—H6···S2	0.93	2.70	3.202 (2)	115
C12—H12···S1	0.93	2.70	3.199 (2)	115
